# The Effects of Prediction on the Perception for Own-Race and Other-Race Faces

**DOI:** 10.1371/journal.pone.0114011

**Published:** 2014-11-25

**Authors:** Guangming Ran, Qi Zhang, Xu Chen, Yangu Pan

**Affiliations:** 1 Faculty of Psychology, Southwest University (SWU), Chongqing, 400715, China; 2 Research Center of Mental Health Education, Southwest University (SWU), Chongqing, 400715, China; 3 School of Education Science, Guizhou Normal University (GNU), Guizhou, 550001, China; Liaoning Normal University, China

## Abstract

Human beings do not passively perceive important social features about others such as race and age in social interactions. Instead, it is proposed that humans might continuously generate predictions about these social features based on prior similar experiences. Pre-awareness of racial information conveyed by others' faces enables individuals to act in “culturally appropriate” ways, which is useful for interpersonal relations in different ethnicity groups. However, little is known about the effects of prediction on the perception for own-race and other-race faces. Here, we addressed this issue using high temporal resolution event-related potential techniques. In total, data from 24 participants (13 women and 11 men) were analyzed. It was found that the N170 amplitudes elicited by other-race faces, but not own-race faces, were significantly smaller in the predictable condition compared to the unpredictable condition, reflecting a switch to holistic processing of other-race faces when those faces were predictable. In this respect, top-down prediction about face race might contribute to the elimination of the other-race effect (one face recognition impairment). Furthermore, smaller P300 amplitudes were observed for the predictable than for unpredictable conditions, which suggested that the prediction of race reduced the neural responses of human brains.

## Introduction

In recent years, one of the topics of interest within face processing research has been the perception of own-race and other-race faces [1]. The popularity of this topic may be because of the fact that it is the key for understanding the controversial and complex phenomena such as the origin and development of racial prejudices.

Regarding electrophysiological studies on the perception of face race, a wealth of data is available. It has been shown in an old/new recognition paradigm that no effects of face race were observed on the amplitudes of P100 [2]–[4]. P100 component is assumed to reflect early visual processing and is sensitive to basic visual attributes of the stimuli such as luminance and contrast. Following P100, a negative deflection peaking between 130 and 190 ms over occipito-temporal sites is usually observed in experiments using faces as stimuli [5],[6]. This so-called N170 is larger for faces compared to other stimulus categories [5]. Although some reports did not detect effects of face race on N170 amplitudes [4],[7], several other studies reported more negative amplitudes for other-race relative to own-race faces [8],[9]. These discrepancies are probably due to differences in experimental task and stimuli. In addition, race effects were observed in P300, a component responding to motivationally-significant events [10].

To date, none of the previous studies, however, has directly investigated the prediction effect on face race. Considered from the viewpoint of proactive brain [11],[12], human beings do not passively perceive important social features about others such as race and age, but rather continuously generate a set of predictions about them. Given that top-down predictions allow our brains to quickly interpret sensory information by deducing plausible interpretations from noisy and ambiguous data [13]–[16], it is likely that the prediction about race exerts an influence on the subsequent recognition of face stimuli.

Considerable research has indicated that own-race faces are perceived in a configural/holistic manner while other-race faces involve mostly feature-based processing [3],[17]. This might be explained by the fact that individuals were experts at distinguishing between own-race faces but relatively in-experts (novices) at distinguishing between other-race faces [17]. A recent study concluded that prediction could facilitate the perception of visual stimuli that involved mostly feature-based processing [18], suggesting that individuals might employ expertise-like skills to perceive other-race faces when those faces were predictable. It should be noted that individuals acquire expertise-like skills via a mechanism other than accruing actual experience discriminating between other-race faces [19]. In this instance, configural processing is assumed to be increased for predictable other-race faces, which may result in accurate encoding of those faces. In the cross-cultural literature, it was commonly thought that decreased N170 amplitudes reflected the configural processing of faces [2],[3]. Thus, we would predict to find that the N170 amplitudes elicited by Caucasian (other-race) faces, but not Chinese (own-race) faces were significantly smaller in the predictable condition compared to the unpredictable condition.

In addition, some other studies [20],[21] suggested that pre-awareness of incoming events facilitated reallocating of cognitive resources as well as pre-preparation of behavioral coping strategies. Importantly, if that is true, individuals were able to better cope with potential dangers in the environment [21], which implied that less motivated attention might be allocated to faces with a predictable onset than to faces without a predictable onset. We therefore expected to observe smaller P300 amplitudes in the predictable condition.

In the present work, we explored, for the first time to our knowledge, the effects of prediction on the perception for own-race and other-race faces. To do so, we adopted a variation of the S1–S2 paradigm where participants' perception was biased by a face [21],[22]. Previous researchers (e.g., [23]) employed the S1–S2 paradigm to investigate the perceptual bias for own-race faces. This bias has been also observed by using a similar task in which an image of a target face was briefly presented and then the perceiver should later select which of two images matches the target [24],[25].

## Methods

### Participants

Twenty-four Chinese volunteers (13 women and 11 men; mean age  =  20.44, *SD* = 1.21) took part in the experiment and reported no direct contact with members of other-racial (Caucasian) groups. In the city where the study was carried out, 95% of the population was Chinese. All participants were right-handed, with normal or corrected-to-normal vision, and none suffered from affective, psychiatric or neurological disorders. One participant was excluded due to the high level of trait anxiety.

### Ethics statement

The ethics committee of Southwest University of China approved this study and the recruitment of participants. Written informed consent was obtained from all participants prior to conducting formal experiment. The experimental procedure was conducted in accordance with the Helsinki guidelines as per the World Health Organization [26].

### Materials

Materials consisted of 8 house and 108 face images (4 cue faces and 104 target faces). Each face portrayed a neutral expression (mean valence: 4.59±0.84, [9-point scale]). Half of the face images were Caucasian faces, and the other half were Chinese ones. Caucasian faces (50% female) were taken from the MUG Facial Expression database [27], whereas Chinese faces (50% female) were selected from the Chinese Facial Affective Picture System database [28]. The mean attractiveness for Caucasian and Chinese faces was comparable (Caucasian: 3.54±0.84, Chinese: 3.74±0.53; F(1, 103)  = 2.17; P = 0.144, [9-point scale]). All images were identical in size, background, spatial frequency, contrast and brightness. The viewing angle of each image was 2.8×3.7°, with a screen of 72 pixels per inch. The experiment comprised three blocks, yielding a total of 256 trials. The onset sequence of the four conditions was randomized across the trials.

### Procedure

**Figure 1:**
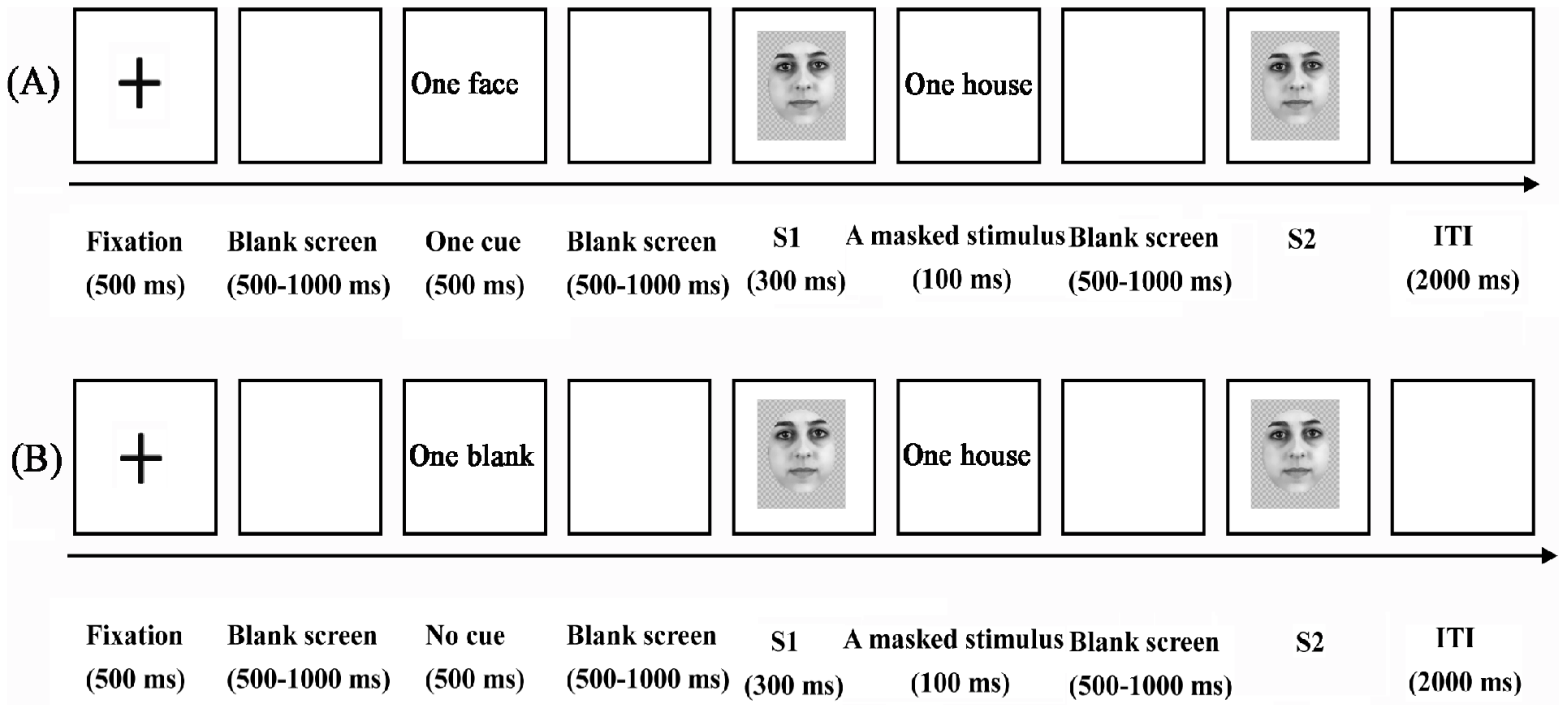
Schematic illustration of the experimental procedure.

Participants were seated comfortably 90 cm from the computer screen and were instructed to try their best to avoid head movements and eye blinks. On each trial of the experiment, a black fixation cross was first shown for 500 ms, after which the cross disappeared and the screen remained empty for 500–1000 ms. Next, either a blank screen (unpredictable condition) or a cue face (predictable condition, indicating the race of the faces to be seen) was depicted for 500 ms. After 500–1000 ms, the first target face (S1) appeared for 300 ms. Following the S1, a masked stimulus (one house image) was presented for 100 ms and subsequently the screen remained empty for 500–1000 ms. At last, the second target face (S2) was presented until a response was made [29],[30]. Participants were asked to judge whether the second target face (S2) had the same or a different identity to the first target face (S1). A schematic illustration of the procedure is shown in Figure 1 (A) and (B). Before the experiment, participants performed 10 practice trials to familiarize themselves with the experimental procedure. Previous studies reported that results using the null cue (a visual stimulus without informative about the valence of the stimulus) as the unpredictable condition were similar to those using the no cue (a blank white screen) [31].

### Electrophysiological recording and analysis

The electroencephalography (EEG) was recorded by a BrainAmps system (Brain Products, Munchen, Germany) from 64 scalp sites according to the 10–20 system positions with a reference at FCz [2],[32]. A common average reference was recalculated. The vertical electrooculogram (EOG) was recorded with electrodes placed below the right eye and the horizontal EOG was recorded from the right orbital rim. Electrode impedance was maintained below 5 kΩ. Both the EEG and EOG activities were amplified using a DC-100 Hz bandpass and continuously sampled at 500 Hz/channel. The mean and range of trial numbers after artifact rejection in each condition are listed in Table 1.

**Table 1:** Mean and range of trial numbers after artifact rejection in each condition.

	Unpredictable Caucasian	Unpredictable Chinese	Predictable Caucasian	Predictable Chinese
**Mean**	57.17	58.08	58.25	57.83
**Range**	44–64	50–63	50–64	50–63

For ERP analysis, EEG activities for correct responses in each condition were separately averaged. On the basis of previous studies [33]–[35], the P100 component (80–140 ms) was determined over O1/O2 and PO3/PO4 electrodes, and the N170 component (130–190 ms) was determined over P5/P6, P7/P8, and PO7/PO8 electrodes. Peak amplitudes and latencies of these two components were subjected to repeated-measure ANOVAs with prediction (predictable vs. unpredictable), stimulus race (Caucasian vs. Chinese) and hemisphere (right vs. left) as within-participant factors. Furthermore, the P300 (300–500 ms) was observed and quantified as mean amplitudes at CPz, Pz, POz, CP1/CP2, P1/P2, and PO3/PO4 electrodes [36]. The time window for P300 was chosen on the basis of previous experience with experiments on face recognition [37]. Mean amplitudes of this component were entered into a 2×2×3 ANOVA with the within-participant factors prediction, stimulus race and hemisphere (right, middle vs. left). The ERP data were analyzed off-line with BrainVision Analyzer (Brain Products). All degrees of freedom for the F-ratio were corrected according to the Greenhouse–Geisser method.

We finally carried out correlation analyses to assess the relationship between the behavioral magnitude of the facilitated (predictable) effect and the electrophysiological magnitude of the reduced neural responses. The index of the magnitude of facilitated effect was calculated by subtracting the accuracy reaction times in predictable condition to those in unpredictable condition for own-race and other-race faces of each participant. Similarly, the electrophysiological magnitude of the reduced neural responses was obtained by computing the differences of ERP amplitudes between predictable and unpredictable conditions.

## Results

### Behavioral results

An ANOVA for correct reaction times yielded a main effect for prediction (*F*(1, 23)  = 7.68, *P* = 0.011, η^2^_p_ = 0.25), indicating that participants responded faster to predictable faces (*M*±*SD*, 647.75±121.60 ms) than to unpredictable ones (669.00±145.52 ms). However, there was no main effect of stimulus race, and no prediction × stimulus race interaction (*P*>0.05 in both cases). The corresponding ANOVA for accuracy rates did not yield any significant effects (*P*>0.05).

### Electrophysiological results

#### P100

The analysis of P100 latency yielded no significant main effects (*stimulus race*: *F*(1, 23)  = 2.89, *P* = 0.103; *prediction*: *F*(1, 23)  = 0.02, *P* = 0.886; *hemisphere*: *F*(1, 23)  = 2.12, *P* = 0.159) or interactions (*hemisphere × stimulus race*: *F*(1, 23)  = 2.91, *P* = 0.102; *hemisphere × prediction*: *F*(1, 23)  = 0.95, *P* = 0.340; *prediction × stimulus race*: *F*(1, 23)  = 0.75, *P* = 0.396). The ANOVA for P100 peak amplitude resulted in a marginal interaction between prediction and stimulus race (*F*(1, 23)  = 3.57, *P* = 0.072, η^2^_p_ = 0.13). No other significant amplitude differences were observed on this component (*P*>0.05 for all).

#### N170

**Figure 2:**
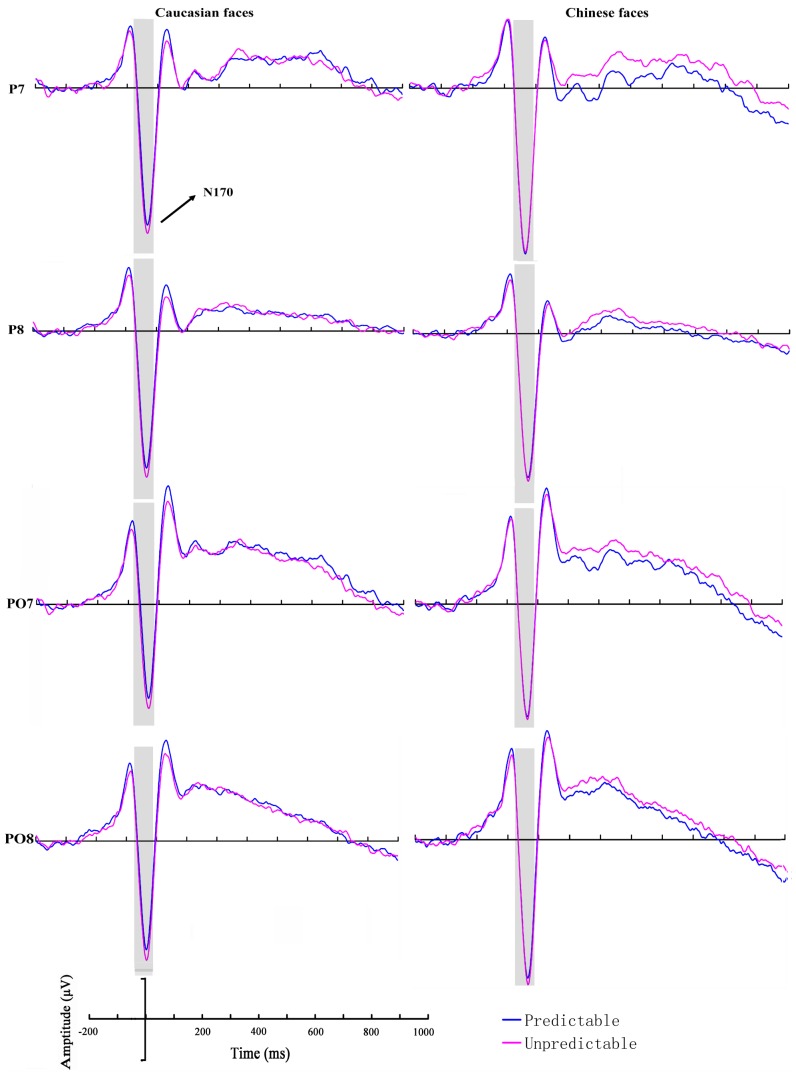
Grand-averaged event-related brain potentials (ERPs) recorded at P7, P8, PO7 and PO8 in response to Caucasian and Chinese faces for predictable and unpredictable conditions.

The ANOVA of N170 latency revealed a significant main effect of stimulus race (*F*(1, 23) 11.96, *P* = 0.002, η^2^_p_ = 0.34), with longer latencies for Caucasian (166.81±9.31 ms) in comparison to Chinese faces (165.51±9.11 ms). The analysis on N170 peak amplitude revealed a significant main effect of prediction (*F*(1, 23)  = 5.95, *P* = 0.023, η^2^_p_ = 0.21), with smaller amplitudes for predictable (−8.57±5.59 µV) compared to unpredictable stimuli (−8.97±5.56 µV), and a main hemisphere effect (*F*(1, 23)  = 9.50, *P* = 0.005, η^2^_p_ = 0.29), with larger amplitudes over the right hemisphere electrodes (*left*: −7.76±5.44 µV; *right*: −9.88±6.27 µV). More importantly, the interaction between prediction and stimulus race was significant (*F*(1, 23)  = 4.79, *P* = 0.039, η^2^_p_ = 0.17). The amplitudes elicited by Caucasian faces, but not Chinese faces were found to be significantly smaller in the predictable condition compared to the unpredictable condition (*Predictable Caucasian*: −8.34±5.70 µV, *Unpredictable Caucasian*: −9.06±5.80 µV, *P* = 0.005) (Figure 2).

#### P300

**Figure 3:**
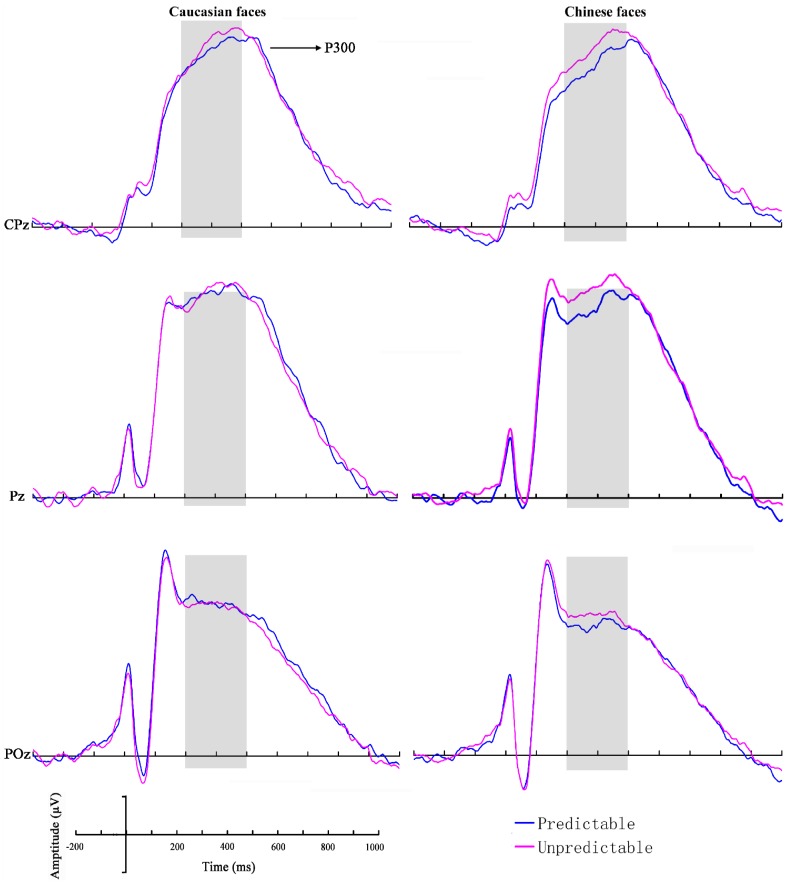
Grand-averaged ERPs recorded at CPz, Pz and POz in response to Caucasian and Chinese faces for predictable and unpredictable conditions.

**Figure 4:**
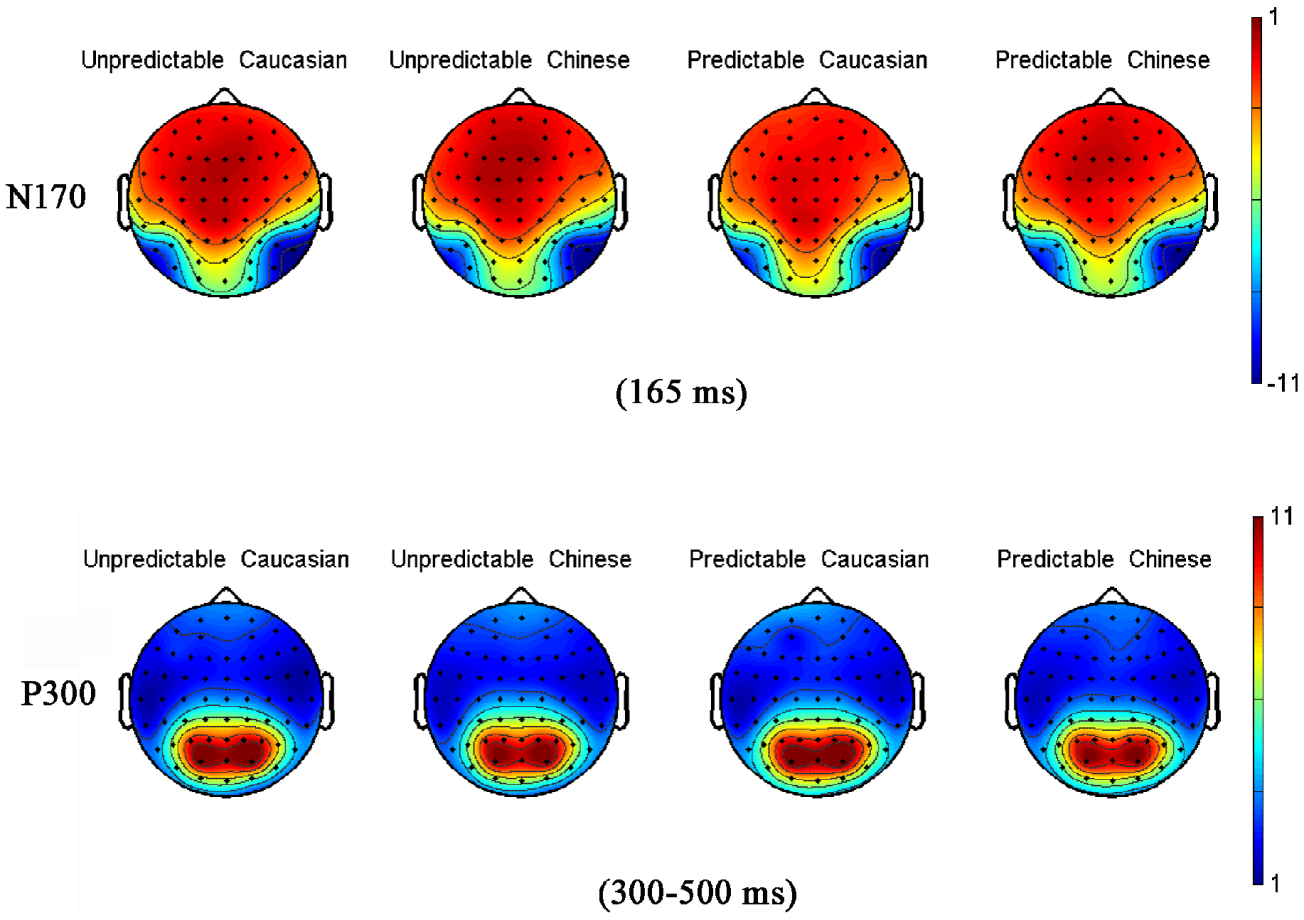
Grand-averaged ERP topographies of the N170 and P300 components in each condition.

For the P300 mean amplitudes, a significant main effect for stimulus race (*F*(1, 23)  = 16.90, *P*<0.001, η^2^_p_ = 0.42), reflecting larger amplitudes for Caucasian faces, was detected (*Caucasian*: 8.94±4.14 µV; *Chinese*: 8.41±4.00 µV). Crucially, the main effect of prediction reached significance (*F*(1, 23)  = 4.90, *P* = 0.037, η^2^_p_ = 0.18), which was due to smaller amplitudes for the predictable stimuli (8.50±4.11 µV) than for the unpredictable stimuli (8.85±4.03 µV) (Figure 3). Furthermore, unlike the N170 component, the P300 showed no significant main effect of hemisphere [*F*(2, 38)  = 1.87, *P* = 0.165, η^2^_p_ = 0.08]. There were also not any significant interactions (all *P*>0.05). The grand average ERP topographies of the N170 and P300 components in each condition are shown in Figure 4.

### Behavioral and electrophysiological results

**Figure 5:**
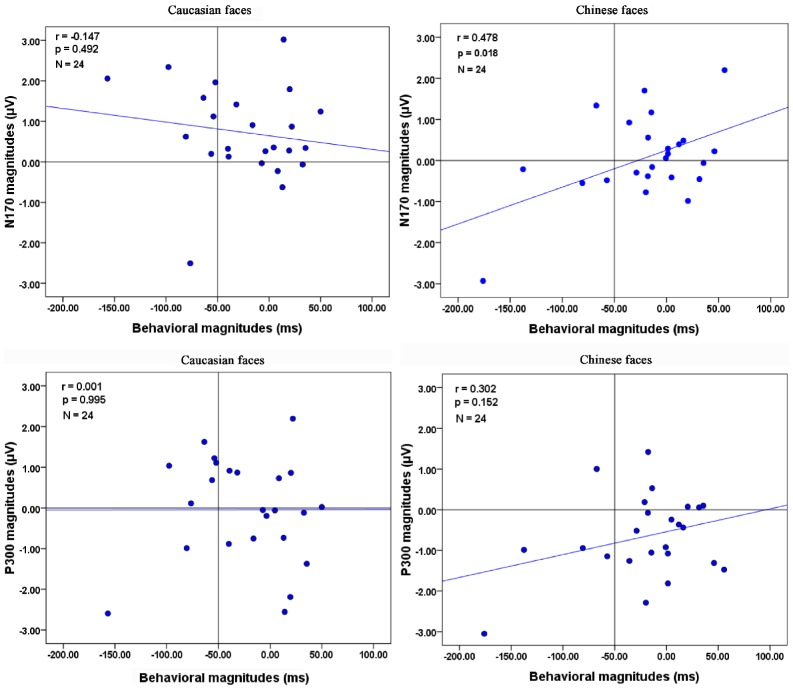
Correlations between the behavioral magnitude of the facilitated effect and the electrophysiological magnitude of the reduced neural responses. Pearson coefficient, respective p values and number of participants are reported in the top left corner.

As demonstrated in Figure 5, a significant correlation was observed between the behavioral magnitude of the facilitated effect and the N170 magnitude of the reduced neural responses only when the participants viewed Chinese faces (*Chinese*: *r(24)*  = 0.478, *P* = 0.018; *bootstrap confidence interval* (CI)  =  [−0.272, −0.789]; *Caucasian*: *r(24)*  = −0.147, *P* = 0.492; CI =  [−0.619, 0.369]). Moreover, while the same pattern of results was observed for the relationship between behavioral data and P300 amplitudes, the correlations were non significant (*Chinese*: *r(24)*  = 0.302, *P* = 0.152, (CI)  =  [−0.303, −0.684]; *Caucasian*: *r(24)*  = 0.001, *P* = 0.995; CI =  [−0.602, −0.500]).

## Discussion

In the present study, we employed a facial discrimination task where perception was biased by one cue to investigate how top-down prediction influenced the own-race and other-race face processing. Behaviorally, we reported a facilitated effect whereby participants responded faster to predictable than unpredictable faces. Crucially, the current electrophysiological results showed that the N170 amplitudes elicited by other-race faces, but not own-race faces, were found to be significantly smaller in the predictable condition compared to the unpredictable condition. Moreover, smaller amplitudes were observed for the P300 component in the predictable condition, regardless of the races of the faces. Finally, when participants viewed own-race faces, the magnitude of the facilitated effect on accuracy RTs were positively correlated with the magnitude of the reduced neural responses on N170 component.

In the current study, both own-race and other-race faces were processed faster in the predictable condition than the unpredictable condition, which indicates that prior prediction of race speeded up the subsequent perception of face stimuli. This facilitation might be due to the fact that the prediction allowed our brains to quickly deduce plausible interpretations from noisy and ambiguous data, thereby reducing the amount of possible representations of faces that need to be considered [38]–[40]. However, no significant differences between recognizing own-race and other-race faces were observed at a behavioral level. The lack of behavioral evidence of the other-race effect might be due to the limited sample size [41].

As reported by Stahl, Wiese, and Schweinberger [3], other-race faces elicited larger N170 amplitudes and longer latencies, suggesting more feature-based processing for other-race compared with own-race faces. The present data showed that the N170 amplitudes elicited by other-race faces were significantly smaller in the predictable condition compared to the unpredictable condition, a result which might reflect a switch to holistic processing of other-race faces when those faces are predictable.

According to the predictive coding framework [42], predictive encoding strategies in human brain were assumed to be parsimonious (concise). In other words, the brain did not need to devote abundant cognitive resources to maintaining the same information at different levels of the processing hierarchy when it continuously predicted the immediately relevant future. In this way, the resources needed for representing perceptual input are minimized [42],[43]. Given that other-race faces were perceived in a feature-based manner that was not parsimonious processing [18],[44], it could be the case that individuals employ one parsimonious processing manner (a configural/holistic processing style) to identify other-race faces when they continuously predict the races of those faces based on prior similar experiences.

Another potential explanation for the switch to holistic processing is the success in allocating more processing resources for individuating information of other-race faces. It has previously been shown that individuals devote more resources to race-specific information of other-race faces than their individuating information, leading to an inferior recognition performance with other-race faces [45]. However, it seems plausible to suggest that more attentional resources might be allocated to individuating information of other-race faces when the races of those faces are predictable, because the race-specific information of other-race faces has been encoded before the actual occurrences of those faces [18],[19]. As noted explicitly by Hugenberg and colleagues [19], when engaging in the individuating information of face stimuli, observers might employ expertise-like skills to perform perceptual processing. It is therefore possible that predictable other-race faces are perceived in a configural/holistic manner.

Since lower P300 amplitudes were associated with reduced motivated attention [46],[47], the present results demonstrating smaller P300 amplitudes in the predictable condition might indicate that less resources or attention was needed to process faces with a predictable onset than to faces without a predictable onset. More precisely, prediction of race reduces the neural responses of human brains. A sharpening account of prediction [48] would suggest that top-down prediction about face race increases the gain of prediction error neurons encoding the predictable stimulus feature, leading to a quick resolution of prediction error when the input matches the prediction. This suggests that a large prediction error will not ensue, which may result in a decrease in neural activity.

In this study, both N170 and P300 were modulated by top-down prediction about face race, exhibiting smaller amplitudes for the predictable faces. However, the P100 component was not sensitive to this factor, as demonstrated by the absence of a significant main effect of prediction. This may reflect that prediction starts to influence the perception of own-race and other-race faces after those basic visual analyses reflected in the P100 have been completed.

Interestingly, there was a significant correlation between the behavioral magnitude of the facilitated effect and the N170 magnitude of the reduced neural responses when participants viewed own-race faces, suggesting that while top-down prediction reduced the neural responses of human brains, it improved the recognition efficiency at the behavioral level. This phenomenon may be described more simply as “*less is more*” [40]. However, the correlation was non significant when observers were presented with other-race faces, which was consistent with the work by Vizioli and colleagues [4]. In their study, Western Caucasian subjects, who viewed African-American and Eastern-Asian faces, showed no significant correlation between the magnitude of the face inversion effect (FIE) on the electrophysiological data (N170 amplitudes elicited by inverted minus those elicited by upright faces) and the magnitude of the FIE on the behavioral data.

## Conclusions

Although a wealth of research has examined the race effects of human faces, there is no study that directly investigates the influences of top-down prediction on the own-race and other-race face processing. In the present study, both own-race and other-race faces were processed faster in the predictable condition than the unpredictable condition, indicating that prediction of race speeded up the recognition of human faces. Electrophysiological results in the N170 time range showed that the amplitudes elicited by other-race faces were significantly smaller in the predictable condition than the unpredictable condition. This result reflected a switch to holistic processing of other-race faces when those faces are predictable, which may contribute to the elimination of the other-race effect (one face recognition impairment). There were also smaller P300 amplitudes in the predictable condition, implying that prediction of race dampened brain responses to human faces.

## References

[1] Wheeler A, Anzures G, Quinn PC, Pascalis O, Omrin DS, et al (2011) Caucasian infants scan own-and other-race faces differently. PloS One 6:e18621. 21533235 10.1371/journal.pone.0018621 PMC3076379

[2] Caharel S, Montalan B, Fromager E, Bernard C, Lalonde R, et al (2011) Other-race and inversion effects during the structural encoding stage of face processing in a race categorization task: an event-related brain potential study. International Journal of Psychophysiology 79:266–271. 21055428 10.1016/j.ijpsycho.2010.10.018

[3] Stahl J, Wiese H, Schweinberger SR (2008) Expertise and own-race bias in face processing: an event-related potential study. Neuroreport 19:583–587. 18388743 10.1097/WNR.0b013e3282f97b4d

[4] Vizioli L, Foreman K, Rousselet GA, Caldara R (2010) Inverting faces elicits sensitivity to race on the N170 component: a cross-cultural study. Journal of Vision 10:1–23. 10.1167/10.1.15 20143908

[5] Bentin S, Allison T, Puce A, Perez E, McCarthy G (1996) Electrophysiological studies of face perception in humans. Journal of Cognitive Neuroscience 8:551–565. 20740065 10.1162/jocn.1996.8.6.551 PMC2927138

[6] Tortosa M, Lupiáñez J, Ruz M (2012) Race, emotion and trust: an ERP study. Brain Research 1494:44–55. 23220554 10.1016/j.brainres.2012.11.037

[7] Vizioli L, Rousselet GA, Caldara R (2010) Neural repetition suppression to identity is abolished by other-race faces. Proceedings of the National Academy of Sciences 107:20081–20086. 10.1073/pnas.1005751107 PMC2993371 21041643

[8] Stahl J, Wiese H, Schweinberger SR (2010) Learning task affects ERP-correlates of the own-race bias, but not recognition memory performance. Neuropsychologia 48:2027–2040. 20362599 10.1016/j.neuropsychologia.2010.03.024

[9] Wiese H, Kaufmann JM, Schweinberger SR (2012) The neural signature of the own-race bias: evidence from event-related potentials. Cerebral Cortex 24:826–835. 23172775 10.1093/cercor/bhs369

[10] Ito TA, Bartholow BD (2009) The neural correlates of race. Trends in Cognitive Sciences 13:524–531. 19896410 10.1016/j.tics.2009.10.002 PMC2796452

[11] Bar M (2003) A cortical mechanism for triggering top-down facilitation in visual object recognition. Journal of Cognitive Neuroscience 15:600–609. 12803970 10.1162/089892903321662976

[12] Kveraga K, Boshyan J, Bar M (2007) Magnocellular projections as the trigger of top-down facilitation in recognition. Journal of Cognitive Neuroscience 27:13232–13240. 10.1523/JNEUROSCI.3481-07.2007 PMC6673387 18045917

[13] Bar M (2007) The proactive brain: using analogies and associations to generate predictions. Trends in Cognitive Sciences 11:280–289. 17548232 10.1016/j.tics.2007.05.005

[14] Bar M (2009) The proactive brain: memory for predictions. Philosophical Transactions of the Royal Society B: Biological Sciences 364:1235–1243. 10.1098/rstb.2008.0310 PMC2666710 19528004

[15] Bendixen A, SanMiguel I, Schröger E (2012) Early electrophysiological indicators for predictive processing in audition: a review. International Journal of Psychophysiology 83:120–131. 21867734 10.1016/j.ijpsycho.2011.08.003

[16] Kveraga K, Ghuman AS, Bar M (2007) Top-down predictions in the cognitive brain. Brain and Cognition 65:145–168. 17923222 10.1016/j.bandc.2007.06.007 PMC2099308

[17] Tanaka JW, Kiefer M, Bukach CM (2004) A holistic account of the own-race effect in face recognition: evidence from a cross-cultural study. Cognition 93:B1–B9. 15110726 10.1016/j.cognition.2003.09.011

[18] Ran GM, Chen X, Pan YG, Hu TQ, Ma J (2014) Effects of anticipation on perception of facial expressions. Perceptual & Motor Skills 118:195–209. 24724522 10.2466/24.PMS.118k13w4

[19] Hugenberg K, Miller J, Claypool HM (2007) Categorization and individuation in the cross-race recognition deficit: toward a solution to an insidious problem. Journal of Experimental Social Psychology 43:334–340.

[20] Ziv M, Tomer R, Defrin R, Hendler T (2010) Individual sensitivity to pain expectancy is related to differential activation of the hippocampus and amygdala. Human Brain Mapping 31:326–338. 19790170 10.1002/hbm.20867 PMC6870810

[21] Lin H, Gao H, Ye Z, Wang P, Tao L, et al (2012) Expectation enhances event-related responses to affective stimuli. Neuroscience Letters 522:123–127. 22710007 10.1016/j.neulet.2012.06.022

[22] Onoda K, Okamoto Y, Shishida K, Hashizume A, Ueda K, et al (2007) Anticipation of affective images and event-related desynchronization (ERD) of alpha activity: an MEG study. Brain Research 1151:134–141. 17408598 10.1016/j.brainres.2007.03.026

[23] Ye J, Li Y, Wei L, Tang Y, Wang J (2009) The race effect on the emotion-induced gamma oscillation in the EEG. Tianjin, China: IEEE. pp.1–4.

[24] Sangrigoli S, De Schonen S (2004) Recognition of own-race and other-race faces by three-month-old infants. Journal of Child Psychology and Psychiatry 45:1219–1227. 15335342 10.1111/j.1469-7610.2004.00319.x

[25] Sangrigoli S, Pallier C, Argenti AM, Ventureyra V, De Schonen S (2005) Reversibility of the other-race effect in face recognition during childhood. Psychological Science 16:440–444. 15943669 10.1111/j.0956-7976.2005.01554.x

[26] Gilder S (1964) World medical association meets in Helsinki. British Medical Journal 2:299–300. 20790244 10.1136/bmj.2.5404.299 PMC1815581

[27] Aifanti N, Papachristou C, Delopoulos A (2010) The MUG facial expression database.11th International Workshop on Image Analysis for Multimedia Interactive Services (WIAMIS). Desenzano, Italy: IEEE. pp.1–4.

[28] Wang Y, Luo YJ (2005) Standardization and assessment of college students' facial expression of emotion. Chinese Journal of Clinical Psychology 13:396–398.

[29] Abundis-Gutiérrez A, Checa P, Castellanos MC, Rueda MR (2014) Electrophysiological correlates of attention networks in childhood and early adulthood. Neuropsychologia 57:78–92. 24593898 10.1016/j.neuropsychologia.2014.02.013

[30] Craig BM, Lipp OV, Mallan KM (2014) Emotional expressions preferentially elicit implicit evaluations of faces also varying in race or age. Emotion 14:865–878. 25046242 10.1037/a0037270

[31] Gole M, Schäfer A, Schienle A (2012) Event-related potentials during exposure to aversion and its anticipation: the moderating effect of intolerance of uncertainty. Neuroscience Letters 507:112–117. 22172930 10.1016/j.neulet.2011.11.054

[32] Herrmann M, Schreppel T, Jäger D, Koehler S, Ehlis AC, et al (2007) The other-race effect for face perception: an event-related potential study. Journal of Neural Transmission 114:951–957. 17318308 10.1007/s00702-007-0624-9

[33] Peng M, De Beuckelaer A, Yuan L, Zhou R (2012) The processing of anticipated and unanticipated fearful faces: an ERP study. Neuroscience Letters 526:85–90. 22910612 10.1016/j.neulet.2012.08.009

[34] Williams LM, Palmer D, Liddell BJ, Song L, Gordon E (2006) The ‘when’and ‘where’of perceiving signals of threat versus non-threat. Neuroimage 31:458–467. 16460966 10.1016/j.neuroimage.2005.12.009

[35] Luo W, Feng W, He W, Wang NY, Luo YJ (2010) Three stages of facial expression processing: ERP study with rapid serial visual presentation. Neuroimage 49:1857–1867. 19770052 10.1016/j.neuroimage.2009.09.018 PMC3794431

[36] Tacikowski P, Nowicka A (2010) Allocation of attention to self-name and self-face: an ERP study. Biological Psychology 84:318–324. 20298741 10.1016/j.biopsycho.2010.03.009

[37] Falkenstein M, Hohnsbein J, Hoormann J, Blanke L (1991) Effects of crossmodal divided attention on late ERP components: II. Error processing in choice reaction tasks. Electroencephalography and Clinical Neurophysiology 78:447–455. 1712280 10.1016/0013-4694(91)90062-9

[38] Bar M (2004) Visual objects in context. Nature Reviews Neuroscience 5:617–629. 15263892 10.1038/nrn1476

[39] Hsu YF, Hämäläinen JA, Waszak F (2014) Both attention and prediction are necessary for adaptive neuronal tuning in sensory processing. Frontiers in Human Neuroscience 8:1–9. 24723871 10.3389/fnhum.2014.00152 PMC3972470

[40] Kok P, Jehee JF, de Lange FP (2012) Less is more: expectation sharpens representations in the primary visual cortex. Neuron 75:265–270. 22841311 10.1016/j.neuron.2012.04.034

[41] Kancherla V, Van Naarden Braun K, Yeargin-Allsopp M (2013) Dental care among young adults with intellectual disability. Research in Developmental Disabilities 34:1630–1641. 23501584 10.1016/j.ridd.2013.02.006 PMC4492120

[42] Alink A, Schwiedrzik CM, Kohler A, Singer W, Muckli L (2010) Stimulus predictability reduces responses in primary visual cortex. The Journal of Neuroscience 30:2960–2966. 20181593 10.1523/JNEUROSCI.3730-10.2010 PMC6633950

[43] Van de Cruys S, Wagemans J (2011) Gestalts as predictions-some reflections and an application to art. Gestalt Theory 33:325–344.

[44] Bernstein MJ, Young SG, Hugenberg K (2007) The cross-category effect mere social categorization is sufficient to elicit an own-group bias in face recognition. Psychological Science 18:706–712. 17680942 10.1111/j.1467-9280.2007.01964.x

[45] Sporer SL (2001) Recognizing faces of other ethnic groups: an integration of theories. Psychology, Public Policy, and Law 7:36–97.

[46] Field M, Munafò MR, Franken IH (2009) A meta-analytic investigation of the relationship between attentional bias and subjective craving in substance abuse. Psychological Bulletin 135:589–607. 19586163 10.1037/a0015843 PMC2999821

[47] Nijs IM, Franken IH, Muris P (2009) Enhanced processing of food-related pictures in female external eaters. Appetite 53:376–383. 19647769 10.1016/j.appet.2009.07.022

[48] Lee TS, Mumford D (2003) Hierarchical Bayesian inference in the visual cortex. Journal of the Optical Society of America A 20:1434–1448. 10.1364/josaa.20.001434 12868647

